# Drug-Induced Conditioned Place Preference and Its Practical Use in Substance Use Disorder Research

**DOI:** 10.3389/fnbeh.2020.582147

**Published:** 2020-09-29

**Authors:** Greer McKendrick, Nicholas M. Graziane

**Affiliations:** ^1^Neuroscience Graduate Program, Penn State College of Medicine, Hershey, PA, United States; ^2^Department of Anesthesiology and Perioperative Medicine, Penn State College of Medicine, Hershey, PA, United States; ^3^Departments of Anesthesiology and Perioperative Medicine and Pharmacology, Penn State College of Medicine, Hershey, PA, United States

**Keywords:** conditioned place preference, CPP, drug reward, addiction-like behavior, drugs of abuse, substance use disorder, addiction, rodent model

## Abstract

The conditioned place preference (CPP) paradigm is a well-established model utilized to study the role of context associations in reward-related behaviors, including both natural rewards and drugs of abuse. In this review article, we discuss the basic history, various uses, and considerations that are tied to this technique. There are many potential takeaway implications of this model, including negative affective states, conditioned drug effects, memory, and motivation, which are all considered here. We also discuss the neurobiology of CPP including relevant brain regions, molecular signaling cascades, and neuromodulatory systems. We further examine some of our prior findings and how they integrate CPP with self-administration paradigms. Overall, by describing the fundamentals of CPP, findings from the past few decades, and implications of using CPP as a research paradigm, we have endeavored to support the case that the CPP method is specifically advantageous for studying the role of a form of Pavlovian learning that associates drug use with the surrounding environment.

## Introduction

Conditioned place preference (CPP) was developed as a technique to assess the reinforcing properties of opioid drugs (Rossi and Reid, 1976; Katz and Gormezano, 1979; Mucha and Iversen, 1984). Now, CPP is widely used to test context associations based on the rewarding properties of an unconditioned stimulus in many organisms including, rodents (Lu et al., 2005; Akbarabadi et al., 2018; Cunningham, 2019), flies (Kaun et al., 2011), *C. elegans* (Musselman et al., 2012; Engleman et al., 2018), planaria (Hutchinson et al., 2015; Mohammed Jawad et al., 2018; Adams and Byrne, 2019; Phelps et al., 2019), primates (Wang et al., 2012; Borges et al., 2015; Yan et al., 2015; Wu et al., 2016), and humans (Thewissen et al., 2006; Childs and De Wit, 2009, 2013, 2016). Although a widely used behavioral model, CPP is a complex behavior that incorporates Pavlovian learning, memory, and motivated behaviors. Due to the complexity, CPP findings are often difficult to understand and interpret. The purpose of this review article is to define common terms used throughout the CPP literature, as well as to discuss factors that are likely to contribute to CPP behaviors in mammals. We include a section related to the neurobiology of opioid-induced conditioned place preference and we conclude by discussing how CPP and addiction-like behaviors can be combined experimentally to assess spatial memory involved in affective states, and to provide a quantifiable readout of context/environment-specific drug-seeking.

## The Biological Purpose of Pavlovian Learning and How It Relates to Drug-Induced CPP

CPP is posited to be based on Pavlovian learning which refers to our ability to form relationships between temporally-associated stimuli. This form of learning as stated elegantly by Fanselow and Wassum (2015), has an evolutionary function that enables us to anticipate events and alter our behavior accordingly to promote survival (Fanselow and Wassum, 2015). Pavlovian learning is advantageous to reproduction (Domjan and Gutiérrez, 2019) as it influences hormonal responses (Graham and Desjardins, 1980), sexual performance (Zamble et al., 1985), and attraction (Domjan, 1994). For example, fish or quail exposed to a paired cue while seeing, but not interacting with a female, will have an increased number of offspring (fish) or increased number of sperm production and fertilized eggs (quail) when the cue is presented and the barrier between the male and female is removed (Hollis et al., 1989; Matthews et al., 2007). Additionally, Pavlovian learning prepares us for food consumption such that eating and digestion occur simultaneously. Pavlov showed that our physiological response to a cue associated with food will elicit salivary secretion and this salivary section is food-dependent (Pavlov, 2010). For example, the meat will evoke thick and viscous saliva containing high levels of mucus, while different substances like salt, acid, and mustard will evoke the release of “watery” saliva (Pavlov, 2010). Furthermore, Pavlovian learning prepares us for danger as well as rewards. In fear conditioning, neutral stimuli that become associated with an aversive event will evoke freezing behaviors in rodents (Rescorla, 1968; Fanselow, 1986; Iwata and Ledoux, 1988; Maren, 1999), while stimuli associated with drugs of abuse will evoke homeostatic alterations to counter previously experienced drug-induced changes (see “Opponent Process Theory in the Factors to Consider” Section in Siegel et al., 1982). In terms of substance use disorders, Pavlovian learning is critically important for context-induced relapse where re-exposure to drug-associated contexts evokes strong drug-craving (O’Brien et al., 1986, 1992). To study this form of relapse, one must understand how the brain forms and retains drug-context associations, which can be preclinically modeled using the CPP paradigm.

## Conditioned Place Preference as a Measure of Drug Reward

CPP is used to measure associations formed between a rewarding stimulus (e.g., drug) and a contextual environment (Tzschentke, 2007). The paradigm uses a two or three-compartment apparatus with each compartment displaying distinct contextual characteristics (e.g., wall colors/patterns and floor texture). The CPP model consists of three phases: habituation, conditioning, and post-conditioning (i.e., CPP test). During habituation, animals are given free access to all compartments before they are returned to their home cage. The habituation sessions serve two purposes. First, they expose the animal to the apparatus, which is intended to habituate the animal to the environment, and second, they provide a measure of an animal’s baseline preference for each compartment. Measuring the baseline preference allows the experimenter to perform a biased design [pairing a drug with the least preferred side to avoid ceiling effects when the drug is assigned to an already preferred environment (Cunningham et al., 2003)] or an unbiased design [randomly pairing the drug with a context; advantages and disadvantages of both designs can be found here (Cunningham et al., 2006)] as well as exclude animals based on predefined exclusion criteria (e.g., spending >80% of the time in one compartment). However, implementing a biased or unbiased design is up to the experimenter as evidence suggests, at least with morphine, that there are no differences in the outcome of CPP when employing a biased or unbiased approach (Blander et al., 1984). Conditioning sessions consist of a non-contingent (experimenter administered) injection of vehicle (control) or drug given before placing and confining the animal in a distinct context. Control and drug conditioning sessions occur on the same day (separated by 4–6 h) or on alternating days. These pairings take place one time or over multiple days. During the conditioning session, the drug-context associations become acquired (often referred to as the acquisition phase). Lastly, following conditioning sessions, animals undergo a CPP test where they are again given free access to all compartments and the time spent in the drug-paired side is measured, which provides a measure of CPP expression. Selective administration of test compounds can be used to assess effects on different phases of CPP. Administration prior to the drug-context pairing (i.e., conditioning phase) assesses the test compound’s effects on the acquisition of CPP, while administration prior to the CPP test measures effects of the test compound on the CPP expression. CPP is measured as the total time spent in each context on test day, or as a CPP score. CPP scores are calculated as either: (i) time in the drug-paired context on test day minus time in the drug-paired context during habituation; or (ii) time in the drug-paired context on test day minus time in the vehicle-paired context on test day. Significant increases in time spent in the drug-paired side is associated with the rewarding properties of the drug.

**Figure 1:**
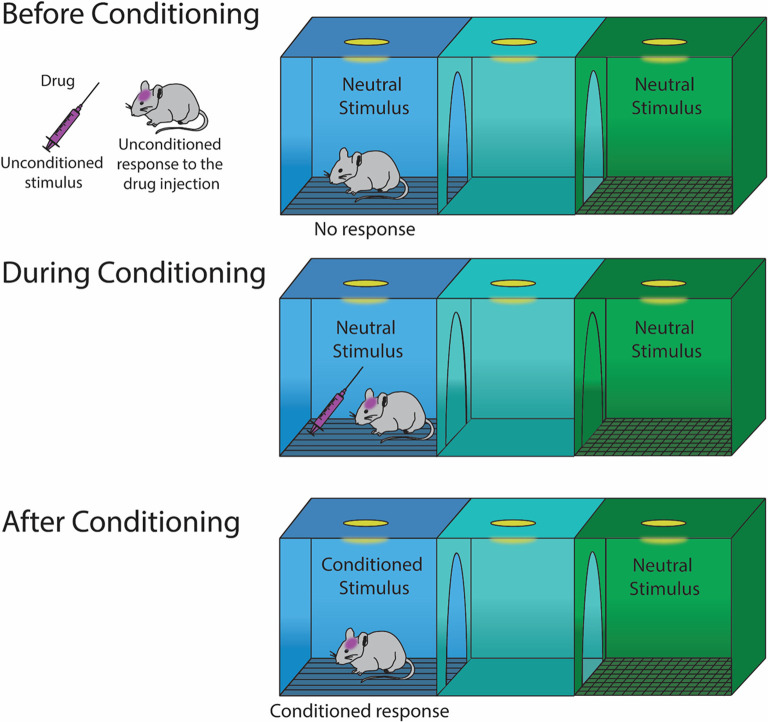
Illustration of Pavlovian learning during conditioning with a drug that elicits hedonic feelings of pleasure. Before conditioning, a drug injection elicits an unconditioned response of pleasure. During conditioning, the neutral stimulus becomes a conditioned stimulus, which results in a conditioned response.

For CPP, in the context of Pavlovian learning, the drug (i.e., the unconditioned stimulus) is expected to elicit a hedonic feeling of pleasure (i.e., an unconditioned response; Figure 1). The drug is paired with a distinct context in the CPP chamber (i.e., a neutral stimulus), which, following conditioning, becomes a conditional stimulus. After conditioning, in the absence of the drug (i.e., the unconditioned stimulus), the drug-paired chamber (i.e., conditional stimulus) is expected to evoke hedonic feelings of pleasure (i.e., conditioned response) leading to approach behaviors toward, and increased time spent in the drug-paired chamber. This approach behavior toward the drug-paired context is similar to sign-tracking behaviors (Huston et al., 2013) which refer to behaviors that are directed toward a stimulus as a result of that stimulus becoming associated with a reward (Huys et al., 2014). Despite this seemingly straightforward behavioral response, there may be many additional underlying factors that contribute to drug-induced CPP. The next section discusses factors that independently and/or synergistically may regulate this complex behavior.

## Factors to Consider When Interpreting Conditioned Place Preference

**Figure 2:**
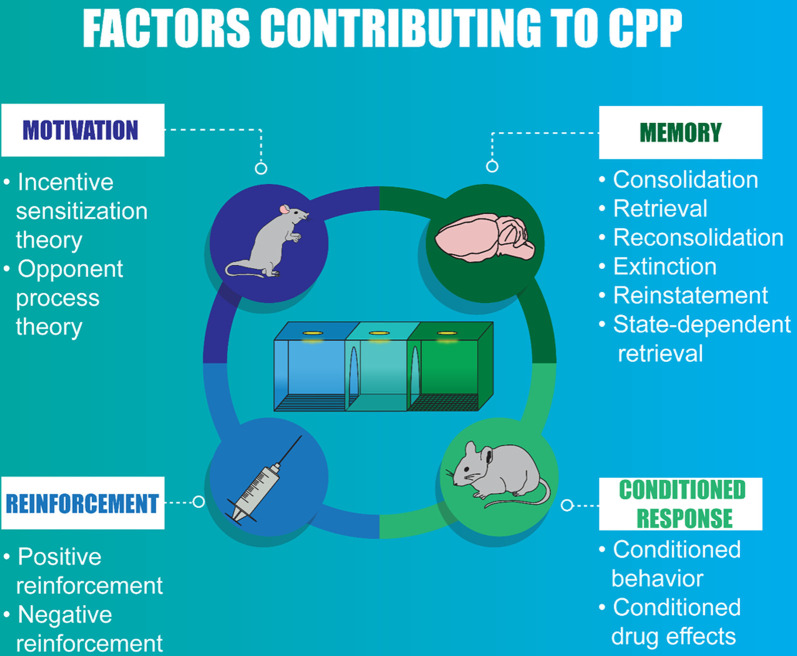
Infographic summarizing the factors that contribute to conditioned place preference (CPP).

As stated above, conditioning in the CPP paradigm refers to pairing a drug with a context. Evidence suggests that a single drug-context pairing (Bardo and Neisewander, 1986; Fenu et al., 2006; Grisel et al., 2014; Nentwig et al., 2017) or repeated drug-context pairings (Cunningham et al., 2006; Dickinson et al., 2009; Ma et al., 2011; Otis and Mueller, 2011; Koo et al., 2014) induces CPP, but these varied exposure protocols may be influenced by different underlying factors including the rewarding properties of the drug, removal of an aversive state, conditioned behaviors, memory, and/or motivated states (Figure 2).

First, a logical interpretation of CPP is that it is mediated by the rewarding properties of the drug. Therefore, the animal seeks out or prefers the drug-paired context during the CPP test because this behavioral response has produced a beneficial, rewarding outcome. This is a valid interpretation and supported by neurobiological responses related to reward encoding that occurs during the conditioning sessions (Tzschentke, 1998, 2007). Additional support comes from human data which not only demonstrate that drug “liking” predicts room liking scores, but also shows the validity of CPP as a translational procedure. In one study, human male and female subjects received either *d*-amphetamine (20 mg) or placebo using a biased design (paired group). Treatments were alternated across successive sessions. A second group received *d*-amphetamine (20 mg) and a placebo in both rooms (unpaired group). The subjective mood was assessed using the Profile of Mood States and participants rated their liking and preference for the testing rooms once before the conditioning sessions and once during re-exposure to the test session after conditioning (Childs and De Wit, 2013). Using this model, Childs and De Wit (2013) showed that the acute positive and negative subjective responses to *d-*amphetamine significantly predicted changes in room liking after conditioning. Additionally, the authors also showed that the context of drug administration can influence acute drug effects on re-administration. This is supported by subjects in the paired group experiencing greater subjective stimulation and drug craving after *d*-amphetamine on the second administration relative to the first (Childs and De Wit, 2013). However, this context-dependent change in subjective drug response is likely drug class-specific as the same authors showed that alcohol effects were consistent across repeated administrations in the same vs. different contexts (Childs and De Wit, 2016). In this latter study, the authors showed that social drinkers developed a place preference for locations paired with alcohol, which was enhanced in subjects experiencing sedative effects from alcohol in those locations (Childs and De Wit, 2016). Therefore, interpreting CPP in rodents as drug reward is validated by human research and is dependent upon the drug, the drug-dose used, and/or the drug-exposure paradigm.

Removal of an aversive state to evoke CPP has been observed in animals expressing chronic pain, in which pairing a pain-relieving drug with a context during conditioning elicits CPP for the drug-paired context (King et al., 2009; Cahill et al., 2013; Navratilova et al., 2013). Additionally, in animals expressing chronic pain, inhibiting the anterior cingulate cortex (ACC), a brain region involved in pain expression, during context pairing elicits CPP for the context paired with ACC inhibition (Gao et al., 2020). These findings suggest that the removal of an aversive state, such as pain, reinforces the animal’s behavioral response to prefer the drug-paired context. Decades of pre-clinical and clinical research have illustrated that, following repeated administration of many drugs of abuse (e.g., opioids, psychostimulants, nicotine, and alcohol), behaviors associated with negative affective states are observed during drug abstinence. These negative states can be physiological, including withdrawal and craving, but also psychological, such as anxiety and depression, and both separately or combined, may be relieved by drug exposure during conditioning, thus facilitating CPP. For example, we found that 5 days of repeated morphine exposure elicits anxiety-like behaviors as well as CPP and that removing the morphine-induced anxiety-like behavior using ketamine is sufficient to block morphine CPP (McKendrick et al., 2020a). Since we did not observe any physiological signs of withdrawal, such as jumping, wet dog shakes, teeth chattering, rearing, tremor, and diarrhea [which coincides with the lack of observed somatic withdrawal symptoms following a more prolonged injection regimen of five daily morphine (10 mg/kg, i.p.) injections over 4 weeks (Robinson and Kolb, 1999)], our results suggest that CPP may be elicited, not only by the rewarding properties of morphine but also by the ability of morphine to relieve “psychological” rather than physiological withdrawal symptoms. There is also evidence that anxiety-like behaviors are linked to somatic withdrawal. Escalating doses of morphine (20–100 mg/kg, i.p.), over 6 days, induce anxiety-like behaviors in the marble burying task, as well as resulting in significant increases in piloerection, jumps, and ptosis (Becker et al., 2017). These studies demonstrate how altering the morphine exposure and concentration paradigm allows the researcher to distinguish morphine-induced negative-affective states from negative affect confounded by somatic signs of withdrawal. In line with this, fewer days of morphine exposure [three morphine injections (10 mg/kg) every other day] does not elicit anxiety-like behaviors (Benturquia et al., 2007), which highlights how the dosing regimen impacts the behavioral paradigm.

Other classes of drug of abuse may also evoke aversive states during conditioning such that the potential “rewarding” effects are mediated by the removal of aversive states. In line with this, pairing a distinct context with an intravenous injection of cocaine during conditioning elicits CPP when the pairing occurs immediately or 5 min after the injection (Ettenberg et al., 1999), however, cocaine-context pairings that occur 15 min after the cocaine injection elicit conditioned place aversion (Ettenberg et al., 1999). Likewise, ethanol, nicotine, and amphetamine exposure show that the immediate effects are rewarding, but that the delayed effects are aversive (Fudala and Iwamoto, 1986, 1987, 1990; Cunningham et al., 1997). Others have shown that, following chronic (14–28 days) non-contingent cocaine administration, rodents displayed less open arm exploration in the elevated plus-maze (Fung and Richard, 1994; Sarnyai et al., 1995; Basso et al., 1999; Rudoy and Van Bockstaele, 2007), which is an indication of the rodent expressing anxiety-like behavior. Furthermore, evidence suggests that repeated, non-contingent cocaine injections (i.e., daily cocaine injections that occur over 5 or 8 days) elicit cocaine-induced anxiety-like behavior when tested on abstinence day 9 or 15 (Valzachi et al., 2013; Hu et al., 2016). Therefore, it is possible that conditioning sessions that occur over many days result in drug-context pairings that alleviate drug-induced negative affective states, subsequently leading to preference for the drug-paired chamber.

Conditioning in the CPP paradigm may also elicit conditioned behavior and conditioned drug effects which, theoretically, may lead to increased or decreased time spent in the drug-paired side during CPP tests (for review, see Huston et al., 2013). Conditioned behaviors, which may be simple or complex, occur spontaneously during conditioning and are inadvertently reinforced during drug exposure, resulting in an increased frequency of the behavior (Skinner, 1948; Staddon and Simmelhag, 1971; Huston et al., 2013). During the test, the drug-paired context may elicit spontaneous behavior (e.g., grooming, rearing, and repetitious movements) and prevent the animal from leaving the conditioned compartment (Huston et al., 2013). Conditioned drug effects refer to drug-induced behavioral responses that become associated with a drug-paired context. After conditioning, re-exposure to the drug-paired context may elicit the reinforced behavior, which may prevent the animal from leaving the drug-paired context, or mask drug-induced CPP (Huston et al., 2013). An example of masked drug-induced CPP is evident from hyperactivity in animals following cocaine administration. This cocaine-induced hyperactivity becomes conditioned to the drug-paired context, which results in conditioned hyperactivity during the CPP test (Saunders et al., 2014). This increased locomotion may increase the probability that the animal leaves the conditioned compartment, thus, inadvertently reducing the true cocaine-induced CPP (Huston et al., 2013).

Memory is another factor to consider that may influence drug-induced CPP. Most CPP tests occur during a period of drug abstinence, so the learned associations that occur during conditioning session/s would have to have been consolidated and maintained for the animal to be able to recall the association when re-exposed to the CPP chamber on test day. Upon drug re-exposure, the memory is retrieved and destabilized, which enables the memory to be updated with new information. Subsequently, the memory is restabilized in a process called reconsolidation (Torregrossa and Taylor, 2013, 2016). Therefore, it is plausible that drug-induced CPP relies on three phases of memory: consolidation, retrieval, and reconsolidation (Milton and Everitt, 2010). Each memory phase is vulnerable to interference in a CPP paradigm, depending upon the time point that the memory interference is initiated by the experimenter. Typically, administering a test compound shortly *after* a conditioning session will assess effects on memory consolidation (Cervo et al., 1997; Hsu et al., 2002; Robinson and Franklin, 2007; Yu et al., 2009). After conditioning is completed, exposing an animal to a test compound just before re-exposure to the CPP apparatus will assess the effects on memory retrieval (Miller and Marshall, 2005; Yim et al., 2006; Fan et al., 2013), and exposing an animal to a test compound following re-exposure to the CPP apparatus will assess the effects on memory reconsolidation (Brown et al., 2007; Otis et al., 2013; Sartor and Aston-Jones, 2014). The timing of test compound administration, if pharmacologically mediated, depends upon the pharmacokinetic properties of the compound. Of note, re-exposure to the CPP apparatus is not the only way to retrieve drug-associated contextual memory as the rewarding properties of the drug may establish state-dependent retrieval (Overton, 1972). In state-dependent retrieval, CPP is more strongly expressed in the presence, vs. the absence, of the drug. This occurs as the learned associations are formed in the presence of the drug during conditioning. Therefore, if the animal learns the associations in a drugged state and performs the test in a drug-free state, retrieval deficits may result due to changes in the internal state of the animal (Spear, 1978; Urcelay and Miller, 2008).

Additionally, CPP memory is liable to extinction and reinstatement. Extinguishing CPP occurs over many days and is often performed by confining the animal to the drug-paired compartment in the absence of the drug, then, on the next day, the animal is given free access to all compartments. This procedure is repeated until the animal reaches extinction criteria (Hearing et al., 2016). CPP is then reinstated with a drug-prime injection or stress exposure (Aguilar et al., 2009).

Reinstatement paradigms are frequently compared to the human experience known as “relapse,” but an important distinction is that relapse in humans is often characterized by a resumption of drug-taking, whereas in rodent models, these reinstatement models are performed in a drug-free state and/or without the ability to continue drug exposure (Sanchis-Segura and Spanagel, 2006). Therefore, it is more accurate to state that reinstatement in CPP more directly reflects a continuation of CPP behaviors, whether it be triggered by a drug-prime injection or a stressor. Types of stressors that have been utilized to trigger reinstatement include: (1) naturalistic stressors, such as water/food deprivation, physical restraint stress (Ribeiro Do Couto et al., 2006), painful stimuli such as the foot-shock paradigm (Wang et al., 2000; Sanchez and Sorg, 2001), and fear/anxiety-inducing stimuli such as the forced swim stress (Sanchez and Sorg, 2001; Ribeiro Do Couto et al., 2006; Redila and Chavkin, 2008); (2) social disruption/conflict stressors including social isolation and maternal deprivation (Ribeiro Do Couto et al., 2006; Calpe-López et al., 2020); and (3) pharmacological stressors, such as injections of agonists of the kappa opioid system (Redila and Chavkin, 2008), and yohimbine (Mantsch et al., 2010). While comparisons of drug-prime vs. stressor-induced reinstatement models are common concerning operant drug self-administration paradigms, they are rather limited in the field of CPP (Mantsch et al., 2016). The findings of Ribeiro Do Couto et al. (2006) demonstrate that social defeat stress is similar to physical restraint stress at reinstating morphine conditioned place preference. Also, one study by Wang et al. (2000) found that both foot-shock stress and an acute morphine prime injection sufficiently reinstated extinguished morphine conditioned place preference. Therefore, future studies are needed to directly compare CPP reinstatement models.

Motivation may also contribute to increases in time spent in the drug-paired compartment during CPP tests. Evidence for this comes from a study showing that a hungry animal will approach contexts previously associated with food, whereas the same animal, when water-deprived, will approach contexts associated with fluid (Perks and Clifton, 1997). Similarly, with drugs of abuse, morphine-dependent chimpanzees given daily, passive injections of morphine and then trained to choose between a white box hiding a syringe filled with morphine or a black box hiding a banana, will choose the white box when deprived of morphine, and choose the black box when pretreated with their daily dose of morphine (Spragg, 1940). These drug-induced motivated behaviors are potentially explained by the combined incentive sensitization and opponent-process theories of substance use disorders (Koob et al., 1989; Robinson and Berridge, 1993, 2008). Here, the drug of abuse elicits an unnatural, strong hedonic sensation of pleasure resulting in the drug becoming highly salient, attractive, and “wanted” (Robinson and Berridge, 1993). Meanwhile, the brain automatically compensates and dampens drug reward by recruiting opponent processes, which, over time, following repeated drug exposure, become quicker, stronger, and longer-lasting, leading to negative affective states (Solomon and Corbit, 1978; Koob et al., 1989; Koob and Le Moal, 2008; Grisel, 2019). It is possible that, in patients diagnosed with substance use disorders, a reward is required to sufficiently curtail these negative affective states. Given the incentive salience that the nervous system attributes to the act of drug taking, the negative affective state may drive drug craving and the recall of Pavlovian associations related to drug taking, thus directing motivated drug-seeking behaviors (O’Brien, 1975; Perkins and Grobe, 1992; Zinser et al., 1992; Wetter et al., 1994; Cooney et al., 1997; Baker et al., 2004; Conklin and Perkins, 2005; Fox et al., 2007; Wikler, 2013).

When interpreting CPP, considering factors such as the rewarding properties of the drug, alleviation of aversive states, conditioned behavior, conditioned drug effects, memory, and/or motivational states, has the potential to lead to more comprehensive assessments. Additionally, considering how these factors work independently and/or synergistically has the potential to explain drug-specific effects that direct behaviors toward, or away from, a stimulus, and/or the underlying neurobiological mechanisms contributing to the behavior. These factors may be unique to addictive-drug categories (e.g., opioids, psychostimulants, cannabis, dissociative, inhalants, depressants, and hallucinogens), or to addictive drugs vs. natural rewards (Spiteri et al., 2000; Yonghui et al., 2006; Steiner et al., 2013).

## Neurobiology of Drug-Induced CPP: Focus on Opioid CPP

Ongoing research investigates the neurobiological mechanisms that regulate CPP, with evidence supporting the role of the central nervous system in mediating learned associations. Seminal work by Schultz et al. (1997) showed that, in monkeys, dopamine neuron firing occurred directly after a juice reward, but over time, these neurons began to fire upon exposure to a light cue that preceded the reward. Further support comes from studies showing that neuronal activation in the ventral tegmental area (VTA), a brain region where dopamine neurons are expressed, is necessary for the acquisition of morphine CPP (Harris et al., 2004; Moaddab et al., 2009). Additional studies show that lesions of VTA dopaminergic terminals in the ACC block opioid-induced CPP (Narita et al., 2010), while *in vivo* stimulation of VTA dopaminergic projections to the nucleus accumbens enhances morphine CPP (Koo et al., 2012). Additionally, increases in dopamine and dopamine metabolites in the nucleus accumbens are correlated with morphine CPP (Ma et al., 2009), and blocking dopamine receptors in the nucleus accumbens and basolateral amygdala prevents the acquisition of morphine CPP (Fenu et al., 2006; Lintas et al., 2011, 2012). Although evidence suggests that the nucleus accumbens and potentially the VTA are not necessary and sufficient for the acquisition of morphine CPP (shown by lesions in the nucleus accumbens or CPP tests in dopamine-deficient mice; Olmstead and Franklin, 1996; Hnasko et al., 2005), more recent reports show that transient inactivation of the VTA or nucleus accumbens inhibits both acquisition and expression of morphine CPP (Moaddab et al., 2009; Esmaeili et al., 2012).

Further support of the nucleus accumbens and VTA in mediating morphine CPP comes from studies focusing on molecular signaling cascades in these brain regions. For example, evidence suggests that the activation of p38 mitogen-activated protein kinase (MAPK) and the transcription factor, nuclear factor-κB (NF-κB) in the nucleus accumbens is critically involved in the acquisition of morphine CPP and that this signaling cascade potentially relies upon the activation of transient receptor potential vanilloid type 1 channel (TRPV1; Zhang et al., 2011, 2012; Hong et al., 2017). Furthermore, antagonizing the transcription factor, ΔFosB in dynorphin-expressing medium spiny neurons, which are putative dopamine D1receptor-expressing medium spiny neurons (McDevitt and Graziane, 2018), or inhibiting the transcription factor cAMP response element (CRE)-binding protein (CREB) in the nucleus accumbens, decreases and increases morphine CPP, respectively (Nestler et al., 2001; Barrot et al., 2002; Zachariou et al., 2006). Lastly, the inhibition of phosphodiesterase (PDE) 10A, which inhibits cAMP- and cGMP-mediated intracellular signaling and is selectively expressed in the nucleus accumbens, inhibits the acquisition of morphine-induced CPP (Mu et al., 2014). In the VTA, inhibition of the mitogen-activated protein kinase kinase (MEK)-extracellular signal-regulated kinase (ERK) pathway blocks the acquisition of morphine CPP (Lin et al., 2010).

Also, there are other brain regions involved in opioid CPP such as the pedunculopontine tegmental nucleus (PPTg), which, when lesioned, blocks morphine-induced CPP (Olmstead and Franklin, 1997; Olmstead et al., 1998). The PPTg sends cholinergic inputs to the VTA, which elicit dopamine-neuron depolarization and increase firing in activated neurons (Floresco et al., 2003). Therefore, it would be expected that the cholinergic neurons of the PPTg are involved in the formation of opioid-context associations *via* activation of dopamine neurons in the VTA. However, evidence suggests that, in heroin-induced CPP, PPTg cholinergic cells that project to the VTA are not involved in opioid CPP (Steidl et al., 2014). Rather, orexin neurons that project from the lateral hypothalamus to the VTA, and the hippocampal dentate gyrus, are critical in the formation of associations between contextual cues and morphine (Harris et al., 2007; Guo et al., 2016) with evidence suggesting that this morphine-induced activation of orexinergic neurons relies on corticotropin-releasing factor 1 receptor (CRF1R) activation in morphine CPP (Lasheras et al., 2015).

Seminal work in the field of fear conditioning supports the role of the hippocampus in mediating contextual encoding (Selden et al., 1991; Kim and Fanselow, 1992; Phillips and Ledoux, 1992) and research in opioid-induced contextual learning suggests that the hippocampus may play a similar role. The activation of cholinergic and dopaminergic systems in the dorsal hippocampus regulates the acquisition of morphine CPP (Rezayof et al., 2003, 2006). Additionally, long-term potentiation (enhanced synaptic transmission) in the CA1 region of the hippocampus, which is associated with learning and memory (Kauer and Malenka, 2007), is disrupted (unknown whether it is blocked or occluded) in rodents expressing morphine CPP (Portugal et al., 2014). *In vivo* electrophysiological studies have shown that long-term potentiation of glutamate transmission at hippocampal ventral subiculum to the nucleus accumbens shell is facilitated in rats following re-exposure to the morphine-paired chamber (Li et al., 2017), with evidence suggesting that this potentiation is involved in spatial learning (Goto and Grace, 2005). Recently, it has been shown that astrocytic μ-opioid receptor activation in the CA1 region of the hippocampus is necessary and sufficient to enhance synaptic transmission at Schaffer collateral to CA1 synapses and that this long-term potentiation leads to the acquisition of contextual memory (Nam et al., 2019).

There are also molecular signaling cascades in the hippocampus involved, in part, in long-term potentiation that are important for the acquisition of morphine CPP. Inhibiting phosphatidylinositol 3-kinase (PI3K) or its downstream target mammalian target of Rapamycin (mTOR) in hippocampal CA3 prevents the acquisition of morphine CPP and inhibits the morphine-induced activation of PI3K-Akt signaling pathway (Cui et al., 2010). Additionally, inhibiting ERK in the ventral hippocampal-medial prefrontal cortical circuit blocks the formation of opiate contextual memory (Wang et al., 2019).

The central amygdala, another brain region involved in fear conditioning (Ciocchi et al., 2010; Goode and Maren, 2019), also influences the acquisition of morphine CPP in this case, through NMDA receptor and dopamine-D1 receptor activation (Zarrindast et al., 2003; Rezayof et al., 2007). Additionally, inhibition of MEK or NMDA receptors in the central amygdala blocks the expression of morphine-induced place preference (Li et al., 2011).

Cortical regions are also involved in the acquisition of morphine-induced Pavlovian learning including viscerosensory regions like the somatosensory cortex and granular insular cortex, which when lesioned, block morphine CPP (Meng et al., 2009; Li et al., 2013). Additionally, blocking NMDA receptors in the prelimbic cortex, a brain region involved in promoting relapse to both fear and drug-seeking (Ma et al., 2014; Goode and Maren, 2019), potentiates the acquisition of morphine CPP, likely mediated by dopamine receptor activation, glutamate release, and basolateral amygdala activation (Bishop et al., 2011). Furthermore, norepinephrine depletion in the medial prefrontal cortex impairs the acquisition of morphine CPP (Ventura et al., 2005).

As evidenced above, morphine CPP is regulated by glutamatergic, cholinergic, and dopaminergic systems. In addition to this, preclinical evidence suggests that morphine-context associations rely on signaling from another neurotransmitter, hormonal, and neuromodulatory systems, including opioid, GABA, norepinephrine, serotonin, cannabinoid, nitric oxide, hypocretin/orexin, neuropeptide S, and cholecystokinin (Tzschentke, 1998, 2007; Le Merrer et al., 2009; Li et al., 2009; Billa et al., 2010; Karimi et al., 2013; Ghavipanjeh et al., 2015; Loureiro et al., 2016; Zhang et al., 2016; Azizbeigi et al., 2019) as well as systems involved in immune function and inflammation (Ghahremani et al., 2006; Zhang et al., 2012; Chen et al., 2017). Additionally, evidence suggests that morphine-induced suppression of endogenous histamine is important for morphine CPP as bilateral lesions of the tuberomammillary nucleus, a brain region that expresses histamine-releasing neurons, potentiated the development of morphine CPP (Gong et al., 2007). Also, activation of scaffolding proteins such as receptor for activated protein kinase C 1 (RACK1) is necessary for morphine CPP (Wan et al., 2011; Liu et al., 2016). Given the rewarding properties of drugs of abuse, it is reasonable to expect that a drug of abuse that elicits hedonic feelings (i.e., pleasant sensations) will activate neurobiological mechanisms that signal reward during each conditioning trial. However, many of the neurobiological mechanisms described above are potentially involved in negative affective states depending upon the drug exposure paradigm, the induction of dependence or tolerance, and/or the drug class used. The suppression of these negative affective states during conditioning may also contribute to CPP.

Negative affect observed during drug abstinence is timed with neurobiological responses that mediate negative affective states (Koob, 2013, 2020). For example, following repeated exposure to morphine, there are increases in norepinephrine-induced modulation of the extended amygdala (Aston-Jones et al., 1999; Delfs et al., 2000; Smith and Aston-Jones, 2008), activation of the amygdalar corticotrophin-releasing factor (CRF) system (Heinrichs et al., 1995; Maj et al., 2003), norepinephrine release in the extended amygdala (Fuentealba et al., 2000; Aston-Jones and Harris, 2004), and decreases in dopamine transmission (Diana et al., 1995). Also, following repeated exposure to cocaine, the lateral habenula, a brain region whose increased activity is correlated with aversive states (Graziane et al., 2018), has increased activation 15 min after repeated cocaine administration (Jhou et al., 2013), with evidence suggesting that this increase in cocaine-induced lateral habenula activation lasts until abstinence day 2 in rodents with a history of cocaine self-administration (Neumann et al., 2014). Additionally, lateral habenula neuronal firing is increased *in vivo* during ethanol-induced conditioned taste aversion (Tandon et al., 2017). Finally, when activated, the dynorphin-κ opioid system produces aversion and dysphoria in humans and in animals (McLaughlin et al., 2003, 2006; Land et al., 2008, 2009; Sirohi and Walker, 2015), with evidence suggesting that this system is activated during drug abstinence, potentially driving drug-induced negative affective states (Mucha and Herz, 1985; Pfeiffer et al., 1986; Wee and Koob, 2010; Chartoff et al., 2012).

Combined, this section highlights how many different brain regions involved in signaling salient cues (VTA and nucleus accumbens), contributing to affective, emotional, and cognitive control (amygdala, insula, prefrontal cortex, and ACC), signaling sensation (somatosensory cortex), and processing spatial information and memory (hippocampus) work together to acquire and maintain drug-context associations. Interestingly, these same brain regions are implicated in processes related to pain (Bushnell et al., 2013; Navratilova and Porreca, 2014) and fear (Goode and Maren, 2019) demonstrating how pathological processing within and between these brain regions can lead to pathological behaviors that are easily differentiated clinically, but influence similar neurocircuit connections, albeit, likely in different ways.

## Conditioned Place Preference Combined With Oral Self-Administration

Substance use disorder is a chronic, relapsing condition that is characterized by specific hallmark behaviors including the difficulty to stop drug use, augmented motivation to seek and take drugs, continued use despite adverse consequences, and high susceptibility to relapse. Addiction-like behaviors, therefore, encompass all aspects of behavior that contribute to these criteria and can be observed in both basic and clinical settings. CPP fundamentally tests the incentive value of contexts, and how environmental conditions contribute to the formation of drug-context associations. The long-term nature of drug context-seeking behavior is evident in our CPP paradigm, in which we observe a robust CPP with 28 days of abstinence following conditioning and drug exposure (McKendrick et al., 2020a). In line with this finding, the motivation to seek a context associated with a drug is seen by the induction of approach behaviors, with CPP tests following conditioning (Aguilar et al., 2009), which can persist for 12 weeks without any additional drug exposure (Mueller et al., 2002). Similar to operant self-administration models, reinstatement following extinction is also reliably shown in CPP. Reinstatement of drug-induced CPP can be induced by both stress (Wang et al., 2002; Aguilar et al., 2009) and drug-primes (Mueller et al., 2002; Aguilar et al., 2009), all with the added component of drug-associated contexts. The ability to reinstate CPP is indicative of persistent drug-associated memories, which likely results in the propensity to elicit drug cravings and/or directs drug-seeking long into abstinence (O’Brien et al., 1986, 1992). Furthermore, work by LeCocq et al. (2020) has advocated the ability of drug-associated contexts to serve as a vital trigger for reinstatement and renewal of extinguished addiction-like behaviors.

Understanding how drugs of abuse become associated with contexts is critical in the study of addiction to deconstruct how contexts influence drug-seeking behaviors, relapse propensity, and treatment success. Through repetitive pairings with drug use, contexts that were previously neutral gain incentive salience, and this intense association can serve to reinforce the cyclical nature of drug-seeking behaviors. In animal models of drug abuse, the ABA renewal paradigm [whereby the subject is conditioned in one context (A), extinguished in another (B), and is then re-exposed to the original context (A)] emphasizes how environmental contexts that have been associated with drug use can directly prompt reinstatement (LeCocq et al., 2020). Clinical research has established that drug-associated contexts promote cue reactivity, elevate craving responses, and are sufficient to elicit context-induced relapse (LeCocq et al., 2020). Recent studies have suggested that a way to improve the discovery of more effective treatments is to accentuate the influence of environmental contexts and their influence on drug-seeking behaviors (Everitt and Robbins, 2005; Aguilar et al., 2009; LeCocq et al., 2020). Therefore, CPP serves as a specialized paradigm that can be exploited just for this purpose.

Seeking the drug-paired context (approach toward and spending more time in the drug-paired context) is not typically considered drug-seeking. This is because drug-seeking behaviors are associated with operant responses for a drug, and occur during an extinction session in a self-administration model [see Marchant et al. (2013) for a description of this model]. Here, an animal that has learned to press a lever or nose poke into an active hole to receive an intravenous drug injection, continues this behavior in the absence of the drug. The amount of lever presses is measured and associated with drug-seeking behavior. In the CPP model, the drug is administered non-contingently in a paired context, so the approach to the context and time spent in the context is not typically considered drug seeking because the drug has not been operantly available during the conditioning session. However, one may argue that drug-seeking requires a motivated response directed toward a context associated with the drug. In this case, drug-context seeking is a critical step in the process of drug-seeking. The problem, thus far, with this argument is that there has not been any direct measure in the CPP paradigm to demonstrate that drug-context seeking is linked to drug-seeking behaviors. Recently, we have developed a novel CPP approach in which mice can consume solutions while confined to either context, thus, enhancing the paradigm by including a voluntary, motivated behavior [see McKendrick et al. (2020b) for details regarding methodology, set-up, and figures illustrating the procedure]. This oral self-administration model can include natural rewards such as sucrose and saccharin solutions, or drug-containing solutions like morphine. When mice are conditioned with a solution of 0.1 mg/ml morphine dissolved in 0.2% saccharin in one chamber and only 0.2% saccharin in the opposing chamber, there is a significant preference for the morphine-paired context on test day. Furthermore, to incorporate instrumental, drug-seeking behaviors with CPP, water bottles were placed in each context on test day. In addition to CPP, morphine-conditioned mice consumed significantly more water on the morphine-paired side, a finding not observed in saccharin controls (McKendrick et al., 2020b). These results suggest that drug-context seeking in the CPP model is not a passive state and is potentially important for directing drug-seeking behavior. As denoted above, this method can expand on traditional CPP paradigms by including a voluntary drug-taking aspect, which allows one to study the importance of learned drug-context associations that are based on distinguishing spatial characteristics represented by distinct environments, similar to human experiences.

## Conclusion

Drug-induced CPP is a Pavlovian-based behavior, used to model the transition of a neutral stimulus to a conditioned stimulus, which drives a conditioned response (i.e., approach behaviors to a drug-paired context). This complex behavior consists of many overlapping components that may work synergistically or independently to drive place preference. Although not considered a gold standard for modeling addiction-like behaviors, CPP provides a valuable tool that can be used to understand how drugs of abuse become associated with environmental contexts, a process which is implicated in context-induced drug craving and relapse (O’Brien et al., 1986, 1992). Additionally, this approach can provide insight into contingency awareness [knowledge that the conditioned stimuli predict the unconditioned stimulus (Grillon, 2002)], which relies on conscious cognitive operations (Dawson and Furedy, 1976; Lovibond and Shanks, 2002). Despite the limitation of non-contingent drug administration, drug-induced CPP provides a measure of motivated approach behaviors toward a drug-associated environment, which is a critical step in drug-seeking behaviors.

## Author Contributions

GM and NG developed the focus of this review article and wrote the manuscript. All authors contributed to the article and approved the submitted version.

## Conflict of Interest

The authors declare that the research was conducted in the absence of any commercial or financial relationships that could be construed as a potential conflict of interest.

## References

[B1] Adams K. Byrne T. (2019). Histamine alters environmental place preference in planaria. Neurosci. Lett. 705, 202–205. 10.1016/j.neulet.2019.04.061 31054331

[B2] Aguilar M. A. Rodríguez-Arias M. Miñarro J. (2009). Neurobiological mechanisms of the reinstatement of drug-conditioned place preference. Brain Res. Rev. 59, 253–277. 10.1016/j.brainresrev.2008.08.002 18762212

[B3] Akbarabadi A. Niknamfar S. Vousooghi N. Sadat-Shirazi M. S. Toolee H. Zarrindast M. R. (2018). Effect of rat parental morphine exposure on passive avoidance memory and morphine conditioned place preference in male offspring. Physiol. Behav. 184, 143–149. 10.1016/j.physbeh.2017.11.024 29174820

[B5] Aston-Jones G. Delfs J. M. Druhan J. Zhu Y. (1999). The bed nucleus of the stria terminalis. A target site for noradrenergic actions in opiate withdrawal. Ann. N Y Acad. Sci. 877, 486–498. 10.1111/j.1749-6632.1999.tb09284.x 10415666

[B4] Aston-Jones G. Harris G. C. (2004). Brain substrates for increased drug seeking during protracted withdrawal. Neuropharmacology 47, 167–179. 10.1016/j.neuropharm.2004.06.020 15464135

[B6] Azizbeigi R. Farzinpour Z. Haghparast A. (2019). Role of orexin-1 receptor within the ventral tegmental area in mediating stress- and morphine priming-induced reinstatement of conditioned place preference in rats. Basic Clin. Neurosci. 10, 373–382. 10.32598/bcn.9.10.130 32231774 PMC7101517

[B7] Baker T. B. Piper M. E. Mccarthy D. E. Majeskie M. R. Fiore M. C. (2004). Addiction motivation reformulated: an affective processing model of negative reinforcement. Psychol. Rev. 111, 33–51. 10.1037/0033-295x.111.1.33 14756584

[B8] Bardo M. T. Neisewander J. L. (1986). Single-trial conditioned place preference using intravenous morphine. Pharmacol. Biochem. Behav. 25, 1101–1105. 10.1016/0091-3057(86)90092-4 3786364

[B9] Barrot M. Olivier J. D. Perrotti L. I. Dileone R. J. Berton O. Eisch A. J.. (2002). CREB activity in the nucleus accumbens shell controls gating of behavioral responses to emotional stimuli. Proc. Natl. Acad. Sci. U S A 99, 11435–11440. 10.1073/pnas.172091899 12165570 PMC123274

[B10] Basso A. M. Spina M. Rivier J. Vale W. Koob G. F. (1999). Corticotropin-releasing factor antagonist attenuates the “anxiogenic-like” effect in the defensive burying paradigm but not in the elevated plus-maze following chronic cocaine in rats. Psychopharmacology 145, 21–30. 10.1007/s002130051028 10445369

[B11] Becker J. A. J. Kieffer B. L. Le Merrer J. (2017). Differential behavioral and molecular alterations upon protracted abstinence from cocaine versus morphine, nicotine, THC and alcohol. Addict. Biol. 22, 1205–1217. 10.1111/adb.12405 27126842 PMC5085894

[B12] Benturquia N. Le Guen S. Canestrelli C. Lagente V. Apiou G. Roques B. P.. (2007). Specific blockade of morphine- and cocaine-induced reinforcing effects in conditioned place preference by nitrous oxide in mice. Neuroscience 149, 477–486. 10.1016/j.neuroscience.2007.08.003 17905521

[B13] Billa S. K. Xia Y. Morón J. A. (2010). Disruption of morphine-conditioned place preference by a δ2-opioid receptor antagonist: study of μ-opioid and δ-opioid receptor expression at the synapse. Eur. J. Neurosci. 32, 625–631. 10.1111/j.1460-9568.2010.07314.x 20626460 PMC3596814

[B14] Bishop S. F. Lauzon N. M. Bechard M. Gholizadeh S. Laviolette S. R. (2011). NMDA receptor hypofunction in the prelimbic cortex increases sensitivity to the rewarding properties of opiates *via* dopaminergic and amygdalar substrates. Cereb. Cortex 21, 68–80. 10.1093/cercor/bhq060 20392811

[B15] Blander A. Hunt T. Blair R. Amit Z. (1984). Conditioned place preference: an evaluation of morphine’s positive reinforcing properties. Psychopharmacology 84, 124–127. 10.1007/bf00432040 6436880

[B16] Borges A. C. Duarte R. B. M. Nogueira L. Barros M. (2015). Temporal and dose-dependent differences in simultaneously-induced cocaine hypervigilance and conditioned-place-preference in marmoset monkeys. Drug Alcohol Depend. 148, 188–194. 10.1016/j.drugalcdep.2015.01.007 25630962

[B17] Brown T. E. Forquer M. R. Cocking D. L. Jansen H. T. Harding J. W. Sorg B. A. (2007). Role of matrix metalloproteinases in the acquisition and reconsolidation of cocaine-induced conditioned place preference. Learn. Mem. 14, 214–223. 10.1101/lm.476207 17353546 PMC1838561

[B18] Bushnell M. C. Ceko M. Low L. A. (2013). Cognitive and emotional control of pain and its disruption in chronic pain. Nat. Rev. Neurosci. 14, 502–511. 10.1038/nrn3516 23719569 PMC4465351

[B19] Cahill C. M. Xue L. Grenier P. Magnussen C. Lecour S. Olmstead M. C. (2013). Changes in morphine reward in a model of neuropathic pain. Behav. Pharmacol. 24, 207–213. 10.1097/fbp.0b013e3283618ac8 23591124

[B20] Calpe-López C. García-Pardo M. P. Martínez-Caballero M. A. Santos-Ortíz A. Aguilar M. A. (2020). Behavioral traits associated with resilience to the effects of repeated social defeat on cocaine-induced conditioned place preference in mice. Front. Behav. Neurosci. 13:278. 10.3389/fnbeh.2019.00278 31998090 PMC6962131

[B21] Cervo L. Mukherjee S. Bertaglia A. Samanin R. (1997). Protein kinases A and C are involved in the mechanisms underlying consolidation of cocaine place conditioning. Brain Res. 775, 30–36. 10.1016/s0006-8993(97)00866-4 9439825

[B22] Chartoff E. Sawyer A. Rachlin A. Potter D. Pliakas A. Carlezon W. A. (2012). Blockade of kappa opioid receptors attenuates the development of depressive-like behaviors induced by cocaine withdrawal in rats. Neuropharmacology 62, 167–176. 10.1016/j.neuropharm.2011.06.014 21736885 PMC3195851

[B23] Chen J.-X. Huang K.-M. Liu M. Jiang J.-X. Liu J.-P. Zhang Y.-X.. (2017). Activation of TLR4/STAT3 signaling in VTA contributes to the acquisition and maintenance of morphine-induced conditioned place preference. Behav. Brain Res. 335, 151–157. 10.1016/j.bbr.2017.08.022 28827130

[B24] Childs E. De Wit H. (2009). Amphetamine-induced place preference in humans. Biol. Psychiatry 65, 900–904. 10.1016/j.biopsych.2008.11.016 19111278 PMC2693956

[B25] Childs E. De Wit H. (2013). Contextual conditioning enhances the psychostimulant and incentive properties of *d*-amphetamine in humans. Addict. Biol. 18, 985–992. 10.1111/j.1369-1600.2011.00416.x 22129527 PMC4242554

[B26] Childs E. De Wit H. (2016). Alcohol-induced place conditioning in moderate social drinkers. Addiction 111, 2157–2165. 10.1111/add.13540 27447940 PMC5226878

[B27] Ciocchi S. Herry C. Grenier F. Wolff S. B. E. Letzkus J. J. Vlachos I.. (2010). Encoding of conditioned fear in central amygdala inhibitory circuits. Nature 468, 277–282. 10.1038/nature09559 21068837

[B28] Conklin C. A. Perkins K. A. (2005). Subjective and reinforcing effects of smoking during negative mood induction. J. Abnorm. Psychol. 114, 153–164. 10.1037/0021-843x.114.1.153 15709822

[B29] Cooney N. L. Litt M. D. Morse P. A. Bauer L. O. Gaupp L. (1997). Alcohol cue reactivity, negative-mood reactivity and relapse in treated alcoholic men. J. Abnorm. Psychol. 106, 243–250. 10.1037/0021-843x.106.2.243 9131844

[B30] Cui Y. Zhang X. Q. Cui Y. Xin W. J. Jing J. Liu X. G. (2010). Activation of phosphatidylinositol 3-kinase/Akt-mammalian target of Rapamycin signaling pathway in the hippocampus is essential for the acquisition of morphine-induced place preference in rats. Neuroscience 171, 134–143. 10.1016/j.neuroscience.2010.08.064 20826199

[B31] Cunningham C. L. (2019). Genetic relationships between ethanol-induced conditioned place aversion and other ethanol phenotypes in 15 inbred mouse strains. Brain Sci. 9:209. 10.3390/brainsci9080209 31434277 PMC6721285

[B32] Cunningham C. L. Ferree N. K. Howard M. A. (2003). Apparatus bias and place conditioning with ethanol in mice. Psychopharmacology 170, 409–422. 10.1007/s00213-003-1559-y 12955296

[B33] Cunningham C. L. Gremel C. M. Groblewski P. A. (2006). Drug-induced conditioned place preference and aversion in mice. Nat. Protoc. 1, 1662–1670. 10.1038/nprot.2006.279 17487149

[B34] Cunningham C. L. Okorn D. M. Howard C. E. (1997). Interstimulus interval determines whether ethanol produces conditioned place preference or aversion in mice. Anim. Learn. Behav. 25, 31–42. 10.3758/bf03199022

[B35] Dawson M. E. Furedy J. J. (1976). The role of awareness in human differential autonomic classical conditioning: the necessary-gate hypothesis. Psychophysiology 13, 50–53. 10.1111/j.1469-8986.1976.tb03336.x 1244630

[B36] Delfs J. M. Zhu Y. Druhan J. P. Aston-Jones G. (2000). Noradrenaline in the ventral forebrain is critical for opiate withdrawal-induced aversion. Nature 403, 430–434. 10.1038/35000212 10667795

[B37] Diana M. Pistis M. Muntoni A. Gessa G. (1995). Profound decrease of mesolimbic dopaminergic neuronal activity in morphine withdrawn rats. J. Pharmacol. Exp. Ther. 272, 781–785. 7853194

[B38] Dickinson S. D. Kashawny S. K. Thiebes K. P. Charles D. Y. (2009). Decreased sensitivity to ethanol reward in adolescent mice as measured by conditioned place preference. Alcohol. Clin. Exp. Res. 33, 1246–1251. 10.1111/j.1530-0277.2009.00950.x 19389188

[B39] Domjan M. (1994). Formulation of a behavior system for sexual conditioning. Psychon. Bull. Rev. 1, 421–428. 10.3758/bf03210946 24203550

[B40] Domjan M. Gutiérrez G. (2019). The behavior system for sexual learning. Behav. Processes 162, 184–196. 10.1016/j.beproc.2019.01.013 30831223

[B41] Engleman E. A. Steagall K. B. Bredhold K.E. Breach M. Kline H. L. Bell R. L.. (2018). Caenorhabditis elegans show preference for stimulants and potential as a model organism for medications screening. Front. Physiol. 9:1200. 10.3389/fphys.2018.01200 30214414 PMC6125605

[B42] Esmaeili M.-H. Sahraei H. Ali-Beig H. Ardehari-Ghaleh M. Mohammadian Z. Zardooz H.. (2012). Transient inactivation of the nucleus accumbens reduces both the expression and acquisition of morphine-induced conditioned place preference in rats. Pharmacol. Biochem. Behav. 102, 249–256. 10.1016/j.pbb.2012.04.015 22580069

[B43] Ettenberg A. Raven M. A. Danluck D. A. Necessary B. D. (1999). Evidence for opponent-process actions of intravenous cocaine. Pharmacol. Biochem. Behav. 64, 507–512. 10548263 10.1016/s0091-3057(99)00109-4

[B44] Everitt B. J. Robbins T. W. (2005). Neural systems of reinforcement for drug addiction: from actions to habits to compulsion. Nat. Neurosci. 8, 1481–1489. 10.1038/nn1579 16251991

[B45] Fan Y. D. Niu H. C. Huma T. Li L. Wang G. M. Xu L. Q.. (2013). Blockage of glucocorticoid receptors during memory acquisition, retrieval and reconsolidation prevents the expression of morphine-induced conditioned place preferences in mice. Dongwuxue Yanjiu 34, E26–E34. 10.3724/sp.j.1141.2013.e01e26 23389984

[B46] Fanselow M. S. (1986). Associative vs. topographical accounts of the immediate shock-freezing deficit in rats: implications for the response selection rules governing species-specific defensive reactions. Learn. Motiv. 17, 16–39. 10.1016/0023-9690(86)90018-4

[B47] Fanselow M. S. Wassum K. M. (2015). The origins and organization of vertebrate Pavlovian conditioning. Cold Spring Harb. Perspect. Biol. 8:a021717. 10.1101/cshperspect.a021717 26552417 PMC4691796

[B48] Fenu S. Spina L. Rivas E. Longoni R. Di Chiara G. (2006). Morphine-conditioned single-trial place preference: role of nucleus accumbens shell dopamine receptors in acquisition, but not expression. Psychopharmacology 187, 143–153. 10.1007/s00213-006-0415-2 16724186

[B49] Floresco S. B. West A. R. Ash B. Moore H. Grace A. A. (2003). Afferent modulation of dopamine neuron firing differentially regulates tonic and phasic dopamine transmission. Nat. Neurosci. 6, 968–973. 10.1038/nn1103 12897785

[B50] Fox H. C. Bergquist K. L. Hong K. I. Sinha R. (2007). Stress-induced and alcohol cue-induced craving in recently abstinent alcohol-dependent individuals. Alcohol. Clin. Exp. Res. 31, 395–403. 10.1111/j.1530-0277.2006.00320.x 17295723

[B51] Fudala P. J. Iwamoto E. T. (1986). Further studies on nicotine-induced conditioned place preference in the rat. Pharmacol. Biochem. Behav. 25, 1041–1049. 10.1016/0091-3057(86)90083-3 3786357

[B52] Fudala P. J. Iwamoto E. T. (1987). Conditioned aversion after delay place conditioning with nicotine. Psychopharmacology 92, 376–381. 10.1007/bf00210847 3114791

[B53] Fudala P. J. Iwamoto E. T. (1990). Conditioned aversion after delay place conditioning with amphetamine. Pharmacol. Biochem. Behav. 35, 89–92. 10.1016/0091-3057(90)90209-z 2315374

[B54] Fuentealba J. A. Forray M. I. Gysling K. (2000). Chronic morphine treatment and withdrawal increase extracellular levels of norepinephrine in the rat bed nucleus of the stria terminalis. J. Neurochem. 75, 741–748. 10.1046/j.1471-4159.2000.0750741.x 10899950

[B55] Fung Y. K. Richard L. A. (1994). Behavioral consequences of cocaine withdrawal in rats. J. Pharm. Pharmacol. 46, 150–152. 10.1111/j.2042-7158.1994.tb03761.x 8021807

[B56] Gao S.-H. Shen L.-L. Wen H.-Z. Zhao Y.-D. Chen P.-H. Ruan H.-Z. (2020). The projections from the anterior cingulate cortex to the nucleus accumbens and ventral tegmental area contribute to neuropathic pain-evoked aversion in rats. Neurobiol. Dis. 140:104862. 10.1016/j.nbd.2020.104862 32251841

[B57] Ghahremani M. H. Eghtesad E. Tahsili-Fahadan P. Sharifzadeh M. Amini M. Tootian Z. (2006). Inhibition of the cyclooxygenase pathway attenuates morphine-induced conditioned place preference in mice. Pharmacol. Biochem. Behav. 85, 356–361. 10.1016/j.pbb.2006.09.002 17049975

[B58] Ghavipanjeh G. R. Pourshanazari A. A. Alaei H. Karimi S. Nejad M. A. (2015). Effects of temporary inactivation and electrical stimulation of the dorsal raphe nucleus on morphine-induced conditioned place preference. Malays. J. Med. Sci. 22, 33–40. 26023293 PMC4438090

[B59] Gong Y. X. Lv M. Zhu Y. P. Zhu Y. Y. Wei E. Q. Shi H.. (2007). Endogenous histamine inhibits the development of morphine-induced conditioned place preference. Acta Pharmacol. Sin. 28, 10–18. 10.1111/j.1745-7254.2007.00470.x 17184577

[B60] Goode T. D. Maren S. (2019). Common neurocircuitry mediating drug and fear relapse in preclinical models. Psychopharmacology 236, 415–437. 10.1007/s00213-018-5024-3 30255379 PMC6373193

[B61] Goto Y. Grace A. A. (2005). Dopaminergic modulation of limbic and cortical drive of nucleus accumbens in goal-directed behavior. Nat. Neurosci. 8, 805–812. 10.1038/nn1471 15908948

[B62] Graham J. M. Desjardins C. (1980). Classical conditioning: induction of luteinizing hormone and testosterone secretion in anticipation of sexual activity. Science 210, 1039–1041. 10.1126/science.7434016 7434016

[B63] Graziane N. M. Neumann P. A. Dong Y. (2018). A focus on reward prediction and the lateral habenula: functional alterations and the behavioral outcomes induced by drugs of abuse. Front. Synaptic Neurosci. 10:12. 10.3389/fnsyn.2018.00012 29896097 PMC5987018

[B64] Grillon C. (2002). Startle reactivity and anxiety disorders: aversive conditioning, context, and neurobiology. Biol. Psychiatry 52, 958–975. 10.1016/s0006-3223(02)01665-7 12437937

[B65] Grisel J. (2019). Never Enough: The Neuroscience and Experience of Addiction. New York, NY: Knopf Doubleday Publishing Group.

[B66] Grisel J. E. Beasley J. B. Bertram E. C. Decker B. E. Duan C. A. Etuma M.. (2014). Initial subjective reward: single-exposure conditioned place preference to alcohol in mice. Front. Neurosci. 8:345. 10.3389/fnins.2014.00345 25408633 PMC4219544

[B67] Guo S. J. Cui Y. Huang Z. Z. Liu H. Zhang X. Q. Jiang J. X.. (2016). Orexin A-mediated AKT signaling in the dentate gyrus contributes to the acquisition, expression and reinstatement of morphine-induced conditioned place preference. Addict. Biol. 21, 547–559. 10.1111/adb.12236 25757577

[B68] Harris G. C. Wimmer M. Byrne R. Aston-Jones G. (2004). Glutamate-associated plasticity in the ventral tegmental area is necessary for conditioning environmental stimuli with morphine. Neuroscience 129, 841–847. 10.1016/j.neuroscience.2004.09.018 15541905

[B69] Harris G. C. Wimmer M. Randall-Thompson J. F. Aston-Jones G. (2007). Lateral hypothalamic orexin neurons are critically involved in learning to associate an environment with morphine reward. Behav. Brain Res. 183, 43–51. 10.1016/j.bbr.2007.05.025 17599478 PMC2030620

[B70] Hearing M. C. Jedynak J. Ebner S. R. Ingebretson A. Asp A. J. Fischer R. A.. (2016). Reversal of morphine-induced cell-type-specific synaptic plasticity in the nucleus accumbens shell blocks reinstatement. Proc. Natl. Acad. Sci. U S A 113, 757–762. 10.1073/pnas.1519248113 26739562 PMC4725472

[B71] Heinrichs S. C. Menzaghi F. Schulteis G. Koob G. F. Stinus L. (1995). Suppression of corticotropin-releasing factor in the amygdala attenuates aversive consequences of morphine withdrawal. Behav. Pharmacol. 6, 74–80. 10.1097/00008877-199501000-00011 11224314

[B72] Hnasko T. S. Sotak B. N. Palmiter R. D. (2005). Morphine reward in dopamine-deficient mice. Nature 438, 854–857. 16341013 10.1038/nature04172

[B73] Hollis K. L. Cadieux E. L. Colbert M. M. (1989). The biological function of Pavlovian conditioning: a mechanism for mating success in the blue gourami (*Trichogaster trichopterus*). J. Comp. Psychol. 103, 115–121. 10.1037/0735-7036.103.2.115

[B74] Hong S.-I. Nguyen T.-L. Ma S.-X. Kim H.-C. Lee S.-Y. Jang C.-G. (2017). TRPV1 modulates morphine-induced conditioned place preference *via* p38 MAPK in the nucleus accumbens. Behav. Brain Res. 334, 26–33. 10.1016/j.bbr.2017.07.017 28734766

[B75] Hsu E. H. Schroeder J. P. Packard M. G. (2002). The amygdala mediates memory consolidation for an amphetamine conditioned place preference. Behav. Brain Res. 129, 93–100. 10.1016/s0166-4328(01)00376-x 11809499

[B76] Hu P. Zhu W. Zhu C. Jin L. Guan Y. Guan X. (2016). Resveratrol fails to affect cocaine conditioned place preference behavior, but alleviates anxiety-like behaviors in cocaine withdrawn rats. Psychopharmacology 233, 1279–1287. 10.1007/s00213-016-4210-4 26790673

[B77] Huston J. P. Silva M. A. Topic B. Müller C. P. (2013). What’s conditioned in conditioned place preference? Trends Pharmacol. Sci. 34, 162–166. 10.1016/j.tips.2013.01.004 23384389

[B78] Hutchinson C. V. Prados J. Davidson C. (2015). Persistent conditioned place preference to cocaine and withdrawal hypo-locomotion to mephedrone in the flatworm planaria. Neurosci. Lett. 593, 19–23. 10.1016/j.neulet.2015.03.021 25778415

[B79] Huys Q. J. M. Tobler P. N. Hasler G. Flagel S. B. (2014). “Chapter 3—The role of learning-related dopamine signals in addiction vulnerability,” in Progress in Brain Research, eds Diana M. Di Chiara G. Spano P. (Elsevier), 31–77. 10.1016/B978-0-444-63425-2.00003-9 24968776

[B80] Iwata J. Ledoux J. E. (1988). Dissociation of associative and nonassociative concomitants of classical fear conditioning in the freely behaving rat. Behav. Neurosci. 102, 66–76. 10.1037/0735-7044.102.1.66 3355660

[B81] Jhou T. C. Good C. H. Rowley C. S. Xu S. P. Wang H. Burnham N. W.. (2013). Cocaine drives aversive conditioning *via* delayed activation of dopamine-responsive habenular and midbrain pathways. J. Neurosci. 33, 7501–7512. 10.1523/JNEUROSCI.3634-12.2013 23616555 PMC3865501

[B82] Karimi S. Azizi P. Shamsizadeh A. Haghparast A. (2013). Role of intra-accumbal cannabinoid CB1 receptors in the potentiation, acquisition and expression of morphine-induced conditioned place preference. Behav. Brain Res. 247, 125–131. 10.1016/j.bbr.2013.03.022 23523958

[B83] Katz R. J. Gormezano G. (1979). A rapid and inexpensive technique for assessing the reinforcing effects of opiate drugs. Pharmacol. Biochem. Behav. 11, 231–233. 10.1016/0091-3057(79)90019-4 504302

[B84] Kauer J. A. Malenka R. C. (2007). Synaptic plasticity and addiction. Nat. Rev. Neurosci. 8, 844–858. 10.1038/nrn2234 17948030

[B85] Kaun K. R. Azanchi R. Maung Z. Hirsh J. Heberlein U. (2011). A Drosophila model for alcohol reward. Nat. Neurosci. 14, 612–619. 10.1038/nn.2805 21499254 PMC4249630

[B86] Kim J. J. Fanselow M. S. (1992). Modality-specific retrograde amnesia of fear. Science 256, 675–677. 10.1126/science.1585183 1585183

[B87] King T. Vera-Portocarrero L. Gutierrez T. Vanderah T. W. Dussor G. Lai J.. (2009). Unmasking the tonic-aversive state in neuropathic pain. Nat. Neurosci. 12, 1364–1366. 10.1038/nn.2407 19783992 PMC3427725

[B88] Koo J. W. Lobo M. K. Chaudhury D. Labonte B. Friedman A. Heller E.. (2014). Loss of BDNF signaling in D1R-expressing NAc neurons enhances morphine reward by reducing GABA inhibition. Neuropsychopharmacology 39, 2646–2653. 10.1038/npp.2014.118 24853771 PMC4207344

[B89] Koo J. W. Mazei-Robison M. S. Chaudhury D. Juarez B. Laplant Q. Ferguson D.. (2012). BDNF is a negative modulator of morphine action. Science 338, 124–128. 10.1126/science.1222265 23042896 PMC3547365

[B90] Koob G. F. (2013). Addiction is a reward deficit and stress surfeit disorder. Front. Psychiatry 4:72. 10.3389/fpsyt.2013.00072 23914176 PMC3730086

[B91] Koob G. F. (2020). Neurobiology of opioid addiction: opponent process, hyperkatifeia, and negative reinforcement. Biol. Psychiatry 87, 44–53. 10.1016/j.biopsych.2019.05.023 31400808

[B92] Koob G. F. Le Moal M. (2008). Review. Neurobiological mechanisms for opponent motivational processes in addiction. Philos. Trans. R. Soc. Lond. B Biol. Sci. 363, 3113–3123. 10.1098/rstb.2008.0094 18653439 PMC2607326

[B93] Koob G. F. Stinus L. Le Moal M. Bloom F. E. (1989). Opponent process theory of motivation: neurobiological evidence from studies of opiate dependence. Neurosci. Biobehav. Rev. 13, 135–140. 10.1016/s0149-7634(89)80022-3 2682399

[B94] Land B. B. Bruchas M. R. Lemos J. C. Xu M. Melief E. J. Chavkin C. (2008). The dysphoric component of stress is encoded by activation of the dynorphin kappa-opioid system. J. Neurosci. 28, 407–414. 10.1523/JNEUROSCI.4458-07.2008 18184783 PMC2612708

[B95] Land B. B. Bruchas M. R. Schattauer S. Giardino W. J. Aita M. Messinger D.. (2009). Activation of the kappa opioid receptor in the dorsal raphe nucleus mediates the aversive effects of stress and reinstates drug seeking. Proc. Natl. Acad. Sci. U S A 106, 19168–19173. 10.1073/pnas.0910705106 19864633 PMC2776420

[B96] Lasheras M. C. Laorden M. L. Milanés M. V. Núñez C. (2015). Corticotropin-releasing factor 1 receptor mediates the activity of the reward system evoked by morphine-induced conditioned place preference. Neuropharmacology 95, 168–180. 10.1016/j.neuropharm.2014.12.021 25556110

[B97] Le Merrer J. Becker J. A. Befort K. Kieffer B. L. (2009). Reward processing by the opioid system in the brain. Physiol. Rev. 89, 1379–1412. 10.1152/physrev.00005.2009 19789384 PMC4482114

[B98] LeCocq M. R. Randall P. A. Besheer J. Chaudhri N. (2020). Considering drug-associated contexts in substance use disorders and treatment development. Neurotherapeutics 17, 43–54. 10.1007/s13311-019-00824-2 31898285 PMC7007469

[B101] Li W. Gao Y.-H. Chang M. Peng Y.-L. Yao J. Han R.-W.. (2009). Neuropeptide S inhibits the acquisition and the expression of conditioned place preference to morphine in mice. Peptides 30, 234–240. 10.1016/j.peptides.2008.10.004 18992779

[B102] Li Y. J. Ping X. J. Qi C. Shen F. Sun L. L. Sun X. W.. (2017). Re-exposure to morphine-associated context facilitated long-term potentiation in the vSUB-NAc glutamatergic pathway *via* GluN2B-containing receptor activation. Addict. Biol. 22, 435–445. 10.1111/adb.12343 26692025

[B100] Li F. Wang X. S. Dai R. P. Zhang J. Y. Zhou X. F. Hao W.. (2011). The activation of NMDA receptor-ERK pathway in the central amygdala is required for the expression of morphine-conditioned place preference in the rat. Neurotox. Res. 20, 362–371. 10.1007/s12640-011-9250-2 21681580

[B99] Li C. L. Zhu N. Meng X. L. Li Y. H. Sui N. (2013). Effects of inactivating the agranular or granular insular cortex on the acquisition of the morphine-induced conditioned place preference and naloxone-precipitated conditioned place aversion in rats. J. Psychopharmacol. 27, 837–844. 10.1177/0269881113492028 23784741

[B103] Lin X. Wang Q. Ji J. Yu L. C. (2010). Role of MEK-ERK pathway in morphine-induced conditioned place preference in ventral tegmental area of rats. J. Neurosci. Res. 88, 1595–1604. 10.1002/jnr.22326 20091775

[B104] Lintas A. Chi N. Lauzon N. M. Bishop S. F. Gholizadeh S. Sun N.. (2011). Identification of a dopamine receptor-mediated opiate reward memory switch in the basolateral amygdala-nucleus accumbens circuit. J. Neurosci. 31, 11172–11183. 10.1523/jneurosci.1781-11.2011 21813678 PMC6623365

[B105] Lintas A. Chi N. Lauzon N. M. Bishop S. F. Sun N. Tan H.. (2012). Inputs from the basolateral amygdala to the nucleus accumbens shell control opiate reward magnitude *via* differential dopamine D1 or D2 receptor transmission. Eur. J. Neurosci. 35, 279–290. 10.1111/j.1460-9568.2011.07943.x 22236063

[B106] Liu L. Zhu J. Zhou L. Wan L. (2016). RACK1 promotes maintenance of morphine-associated memory *via* activation of an ERK-CREB dependent pathway in hippocampus. Sci. Rep. 6:20183. 10.1038/srep20183 26830449 PMC4735742

[B107] Loureiro M. Kramar C. Renard J. Rosen L. G. Laviolette S. R. (2016). Cannabinoid transmission in the hippocampus activates nucleus accumbens neurons and modulates reward and aversion-related emotional salience. Biol. Psychiatry 80, 216–225. 10.1016/j.biopsych.2015.10.016 26681496

[B108] Lovibond P. F. Shanks D. R. (2002). The role of awareness in Pavlovian conditioning: empirical evidence and theoretical implications. J. Exp. Psychol. Anim. Behav. Process 28, 3–26. 10.1037/0097-7403.28.1.3 11868231

[B109] Lu L. Chen H. Su W. Ge X. Yue W. Su F.. (2005). Role of withdrawal in reinstatement of morphine-conditioned place preference. Psychopharmacology 181, 90–100. 10.1007/s00213-005-2207-5 15739075

[B110] Ma Y. Y. Lee B. R. Wang X. Guo C. Liu L. Cui R.. (2014). Bidirectional modulation of incubation of cocaine craving by silent synapse-based remodeling of prefrontal cortex to accumbens projections. Neuron 83, 1453–1467. 10.1016/j.neuron.2014.08.023 25199705 PMC4295617

[B111] Ma Y.-Y. Meng L. Guo C.-Y. Han J.-S. Lee D. Y.-W. Cui C.-L. (2009). Dose- and time-dependent, context-induced elevation of dopamine and its metabolites in the nucleus accumbens of morphine-induced CPP rats. Behav. Brain Res. 204, 192–199. 10.1016/j.bbr.2009.06.017 19539657

[B112] Ma Y.-Y. Yu P. Guo C.-Y. Cui C.-L. (2011). Effects of ifenprodil on morphine-induced conditioned place preference and spatial learning and memory in rats. Neurochem. Res. 36, 383–391. 10.1007/s11064-010-0342-9 21152977

[B113] Maj M. Turchan J. Śmiałowska M. Przewłocka B. (2003). Morphine and cocaine influence on CRF biosynthesis in the rat central nucleus of amygdala. Neuropeptides 37, 105–110. 10.1016/s0143-4179(03)00021-0 12747942

[B114] Mantsch J. R. Baker D. A. Funk D. Lê A. D. Shaham Y. (2016). Stress-induced reinstatement of drug seeking: 20 years of progress. Neuropsychopharmacology 41, 335–356. 10.1038/npp.2015.142 25976297 PMC4677117

[B115] Mantsch J. R. Weyer A. Vranjkovic O. Beyer C. E. Baker D. A. Caretta H. (2010). Involvement of noradrenergic neurotransmission in the stress- but not cocaine-induced reinstatement of extinguished cocaine-induced conditioned place preference in mice: role for β-2 adrenergic receptors. Neuropsychopharmacology 35, 2165–2178. 10.1038/npp.2010.86 20613718 PMC2939933

[B116] Marchant N. J. Li X. Shaham Y. (2013). Recent developments in animal models of drug relapse. Curr. Opin. Neurobiol. 23, 675–683. 10.1016/j.conb.2013.01.003 23374536 PMC3644546

[B117] Maren S. (1999). Neurotoxic basolateral amygdala lesions impair learning and memory but not the performance of conditional fear in rats. J. Neurosci. 19, 8696–8703. 10.1523/jneurosci.19-19-08696.1999 10493770 PMC6783031

[B118] Matthews R. N. Domjan M. Ramsey M. Crews D. (2007). Learning effects on sperm competition and reproductive fitness. Psychol. Sci. 18, 758–762. 10.1111/j.1467-9280.2007.01974.x 17760768

[B119] McDevitt D. S. Graziane N. M. (2018). Neuronal mechanisms mediating pathological reward-related behaviors: a focus on silent synapses in the nucleus accumbens. Pharmacol. Res. 136, 90–96. 10.1016/j.phrs.2018.08.025 30171902

[B120] McKendrick G. Garrett H. Jones H. E. Mcdevitt D. S. Sharma S. Silberman Y.. (2020a). Ketamine blocks morphine-induced conditioned place preference and anxiety-like behaviors in mice. Front. Behav. Neurosci. 14:75. 10.3389/fnbeh.2020.00075 32508606 PMC7253643

[B121] McKendrick G. Garrett H. Tanniru S. Ballard S. Sun D. Silberman Y.. (2020b). A novel method to study reward-context associations paired with drug-seeking behaviors. J. Neurosci. Methods 343:108857. 10.1016/j.jneumeth.2020.108857 32652184

[B122] McLaughlin J. P. Li S. Valdez J. Chavkin T. A. Chavkin C. (2006). Social defeat stress-induced behavioral responses are mediated by the endogenous kappa opioid system. Neuropsychopharmacology 31, 1241–1248. 10.1038/sj.npp.1300872 16123746 PMC2096774

[B123] McLaughlin J. P. Marton-Popovici M. Chavkin C. (2003). Kappa opioid receptor antagonism and prodynorphin gene disruption block stress-induced behavioral responses. J. Neurosci. 23, 5674–5683. 10.1523/JNEUROSCI.23-13-05674.2003 12843270 PMC2104777

[B124] Meng Z. Liu C. Hu X. Ma Y. (2009). Somatosensory cortices are required for the acquisition of morphine-induced conditioned place preference. PLoS One 4:e7742. 10.1371/journal.pone.0007742 19888465 PMC2766828

[B125] Miller C. A. Marshall J. F. (2005). Molecular substrates for retrieval and reconsolidation of cocaine-associated contextual memory. Neuron 47, 873–884. 10.1016/j.neuron.2005.08.006 16157281

[B126] Milton A. L. Everitt B. J. (2010). The psychological and neurochemical mechanisms of drug memory reconsolidation: implications for the treatment of addiction. Eur. J. Neurosci. 31, 2308–2319. 10.1111/j.1460-9568.2010.07249.x 20497475

[B127] Moaddab M. Haghparast A. Hassanpour-Ezatti M. (2009). Effects of reversible inactivation of the ventral tegmental area on the acquisition and expression of morphine-induced conditioned place preference in the rat. Behav. Brain Res. 198, 466–471. 10.1016/j.neures.2009.09.1456 19073220

[B128] Mohammed Jawad R. A. Hutchinson C. V. Prados J. (2018). Dissociation of place preference and tolerance responses to sucrose using a dopamine antagonist in the planarian. Psychopharmacology 235, 829–836. 10.1007/s00213-017-4801-8 29197982 PMC5847079

[B129] Mu Y. Ren Z. Jia J. Gao B. Zheng L. Wang G.. (2014). Inhibition of phosphodiesterase10A attenuates morphine-induced conditioned place preference. Mol. Brain 7:70. 10.1186/s13041-014-0070-1 25252626 PMC4180334

[B130] Mucha R. F. Herz A. (1985). Motivational properties of kappa and mu opioid receptor agonists studied with place and taste preference conditioning. Psychopharmacology 86, 274–280. 10.1007/bf00432213 2994144

[B131] Mucha R. F. Iversen S. D. (1984). Reinforcing properties of morphine and naloxone revealed by conditioned place preferences: a procedural examination. Psychopharmacology 82, 241–247. 10.1007/bf00427782 6425908

[B132] Mueller D. Perdikaris D. Stewart J. (2002). Persistence and drug-induced reinstatement of a morphine-induced conditioned place preference. Behav. Brain Res. 136, 389–397. 10.1016/s0166-4328(02)00297-8 12429400

[B133] Musselman H. N. Neal-Beliveau B. Nass R. Engleman E. A. (2012). Chemosensory cue conditioning with stimulants in a *Caenorhabditis elegans* animal model of addiction. Behav. Neurosci. 126, 445–456. 10.1037/a0028303 22642886 PMC3367381

[B134] Nam M.-H. Han K.-S. Lee J. Won W. Koh W. Bae J. Y. (2019). Activation of astrocytic μ-opioid receptor causes conditioned place preference. Cell Rep. 28, 1154–1166. 31365861 10.1016/j.celrep.2019.06.071

[B135] Narita M. Matsushima Y. Niikura K. Narita M. Takagi S. Nakahara K.. (2010). Implication of dopaminergic projection from the ventral tegmental area to the anterior cingulate cortex in μ-opioid-induced place preference. Addict. Biol. 15, 434–447. 10.1111/j.1369-1600.2010.00249.x 20731628

[B136] Navratilova E. Porreca F. (2014). Reward and motivation in pain and pain relief. Nat. Neurosci. 17, 1304–1312. 10.1038/nn.3811 25254980 PMC4301417

[B137] Navratilova E. Xie J. Y. King T. Porreca F. (2013). Evaluation of reward from pain relief. Ann. N Y Acad. Sci. 1282, 1–11. 10.1111/nyas.12095 23496247 PMC4028681

[B138] Nentwig T. B. Myers K. P. Grisel J. E. (2017). Initial subjective reward to alcohol in Sprague-Dawley rats. Alcohol 58, 19–22. 10.1016/j.alcohol.2016.11.005 28109344 PMC6339560

[B139] Nestler E. J. Barrot M. Self D. W. (2001). ΔFosB: a sustained molecular switch for addiction. Proc. Natl. Acad. Sci. U S A 98, 11042–11046. 10.1073/pnas.191352698 11572966 PMC58680

[B140] Neumann P. A. Ishikawa M. Otaka M. Huang Y. H. Schluter O. M. Dong Y. (2014). Increased excitability of lateral habenula neurons in adolescent rats following cocaine self-administration. Int. J. Neuropsychopharmacol. 18:pyu109. 10.1093/ijnp/pyu109 25548105 PMC4390528

[B142] O’Brien C. P. (1975). Experimental analysis of conditioning factors in human narcotic addiction. Pharmacol. Rev. 27, 533–543. 1223916

[B143] O’Brien C. P. Childress A. R. Mclellan A. T. Ehrman R. (1992). Classical conditioning in drug-dependent humans. Ann. N Y Acad. Sci. 654, 400–415. 10.1111/j.1749-6632.1992.tb25984.x 1632593

[B141] O’Brien C. P. Ehrman R. N. Ternes J. (1986). “Classical conditioning in human opioid dependence,” in Behavioral Analysis of Drug Dependence, ed. Goldberg S. I. (Orlando, FL: Academic), 329–356.

[B144] Olmstead M. C. Franklin K. B. (1997). The development of a conditioned place preference to morphine: effects of lesions of various CNS sites. Behav. Neurosci. 111, 1313–1323. 10.1037/0735-7044.111.6.1313 9438800

[B145] Olmstead M. C. Franklin K. B. J. (1996). Differential effects of ventral striatal lesions on the conditioned place preference induced by morphine or amphetamine. Neuroscience 71, 701–708. 10.1016/0306-4522(95)00486-6 8867042

[B146] Olmstead M. C. Munn E. M. Franklin K. B. Wise R. A. (1998). Effects of pedunculopontine tegmental nucleus lesions on responding for intravenous heroin under different schedules of reinforcement. J. Neurosci. 18, 5035–5044. 10.1523/jneurosci.18-13-05035.1998 9634569 PMC6792563

[B147] Otis J. M. Mueller D. (2011). Inhibition of β-adrenergic receptors induces a persistent deficit in retrieval of a cocaine-associated memory providing protection against reinstatement. Neuropsychopharmacology 36, 1912–1920. 10.1038/npp.2011.77 21544069 PMC3154110

[B148] Otis J. M. Dashew K. B. Mueller D. (2013). Neurobiological dissociation of retrieval and reconsolidation of cocaine-associated memory. J. Neurosci. 33, 1271–1281. 10.1523/jneurosci.3463-12.2013 23325262 PMC3564635

[B149] Overton D. A. (1972). “State-dependent learning produced by alcohol and its relevance to alcoholism,” in The Biology of Alcoholism: Volume 2: Physiology and Behavior, eds Kissin B. Begleiter H. (Boston, MA: Springer US), 193–217.

[B150] Pavlov P. I. (2010). Conditioned reflexes: an investigation of the physiological activity of the cerebral cortex. Ann. Neurosci. 17, 136–141. 10.5214/ans.0972-7531.1017309 25205891 PMC4116985

[B151] Perkins K. A. Grobe J. E. (1992). Increased desire to smoke during acute stress. Br. J. Addict. 87, 1037–1040. 10.1111/j.1360-0443.1992.tb03121.x 1643396

[B1510] Perks S. M. Clifton P. G. (1997). Reinforcer revaluation and conditioned place preference. Physiol Behav. 61, 1–5. 10.1016/s0031-9384(96)00243-0 8976526

[B152] Pfeiffer A. Brantl V. Herz A. Emrich H. (1986). Psychotomimesis mediated by kappa opiate receptors. Science 233, 774–776. 10.1126/science.3016896 3016896

[B153] Phelps B. J. Miller T. M. Arens H. Hutchinson T. Lang K. A. Muckey L. M.. (2019). Preliminary evidence from planarians that cotinine establishes a conditioned place preference. Neurosci. Lett. 703, 145–148. 10.1016/j.neulet.2019.03.024 30890472

[B154] Phillips R. G. Ledoux J. E. (1992). Differential contribution of amygdala and hippocampus to cued and contextual fear conditioning. Behav. Neurosci. 106, 274–285. 10.1037/0735-7044.106.2.274 1590953

[B155] Portugal G. S. Al-Hasani R. Fakira A. K. Gonzalez-Romero J. L. Melyan Z. Mccall J. G.. (2014). Hippocampal long-term potentiation is disrupted during expression and extinction but is restored after reinstatement of morphine place preference. J. Neurosci. 34, 527–538. 10.1523/jneurosci.2838-13.2014 24403152 PMC3870935

[B156] Redila V. A. Chavkin C. (2008). Stress-induced reinstatement of cocaine seeking is mediated by the kappa opioid system. Psychopharmacology 200, 59–70. 10.1007/s00213-008-1122-y 18575850 PMC2680147

[B157] Rescorla R. A. (1968). Probability of shock in the presence and absence of CS in fear conditioning. J. Comp. Physiol. Psychol. 66, 1–5. 10.1037/h0025984 5672628

[B158] Rezayof A. Golhasani-Keshtan F. Haeri-Rohani A. Zarrindast M. R. (2007). Morphine-induced place preference: involvement of the central amygdala NMDA receptors. Brain Res. 1133, 34–41. 10.1016/j.brainres.2006.11.049 17184750

[B159] Rezayof A. Zarrindast M.-R. Sahraei H. Haeri-Rohani A. (2003). Involvement of dopamine receptors of the dorsal hippocampus on the acquisition and expression of morphine-induced place preference in rats. J. Psychopharmacol. 17, 415–423. 10.1177/0269881103174005 14870954

[B160] Rezayof A. Zatali H. Haeri-Rohani A. Zarrindast M.-R. (2006). Dorsal hippocampal muscarinic and nicotinic receptors are involved in mediating morphine reward. Behav. Brain Res. 166, 281–290. 10.1016/j.bbr.2005.08.010 16191443

[B161] Ribeiro Do Couto B. Aguilar M. A. Manzanedo C. Rodríguez-Arias M. Armario A. Miñarro J. (2006). Social stress is as effective as physical stress in reinstating morphine-induced place preference in mice. Psychopharmacology 185, 459–470. 10.1007/s00213-006-0345-z 16555060

[B163] Robinson T. E. Berridge K. C. (1993). The neural basis of drug craving: an incentive-sensitization theory of addiction. Brain Res. Rev. 18, 247–291. 10.1016/0165-0173(93)90013-p 8401595

[B162] Robinson M. J. Franklin K. B. (2007). Effects of anisomycin on consolidation and reconsolidation of a morphine-conditioned place preference. Behav. Brain Res. 178, 146–153. 10.1016/j.bbr.2006.12.013 17239969

[B164] Robinson T. E. Berridge K. C. (2008). Review. The incentive sensitization theory of addiction: some current issues. Philos. Trans. R. Soc. Lond. B Biol. Sci. 363, 3137–3146. 10.1098/rstb.2008.0093 18640920 PMC2607325

[B165] Robinson T. E. Kolb B. (1999). Morphine alters the structure of neurons in the nucleus accumbens and neocortex of rats. Synapse 33, 160–162. 10.1002/(sici)1098-2396(199908)33:2<160::aid-syn6>3.0.co;2-s 10400894

[B166] Rossi N. A. Reid L. D. (1976). Affective states associated with morphine injections. Physiol. Psychol. 4, 269–274. 10.3758/bf03332869

[B167] Rudoy C. A. Van Bockstaele E. J. (2007). Betaxolol, a selective β1-adrenergic receptor antagonist, diminishes anxiety-like behavior during early withdrawal from chronic cocaine administration in rats. Prog. Neuropsychopharmacol. Biol. Psychiatry 31, 1119–1129. 10.1016/j.pnpbp.2007.04.005 17513029 PMC4287233

[B168] Sanchez C. J. Sorg B. A. (2001). Conditioned fear stimuli reinstate cocaine-induced conditioned place preference. Brain Res. 908, 86–92. 10.1016/s0006-8993(01)02638-5 11457434

[B169] Sanchis-Segura C. Spanagel R. (2006). Behavioural assessment of drug reinforcement and addictive features in rodents: an overview. Addict. Biol. 11, 2–38. 10.1111/j.1369-1600.2006.00012.x 16759333

[B170] Sarnyai Z. Bíró E. Gardi J. Vecsernyés M. Julesz J. Telegdy G. (1995). Brain corticotropin-releasing factor mediates ‘anxiety-like’ behavior induced by cocaine withdrawal in rats. Brain Res. 675, 89–97. 10.1016/0006-8993(95)00043-p 7796157

[B171] Sartor G. C. Aston-Jones G. (2014). Post-retrieval extinction attenuates cocaine memories. Neuropsychopharmacology 39, 1059–1065. 10.1038/npp.2013.323 24257156 PMC3957116

[B172] Saunders B. T. O’Donnell E. G. Aurbach E. L. Robinson T. E. (2014). A cocaine context renews drug seeking preferentially in a subset of individuals. Neuropsychopharmacology 39, 2816–2823. 10.1038/npp.2014.131 24896613 PMC4200491

[B173] Schultz W. Dayan P. Montague P. R. (1997). A neural substrate of prediction and reward. Science 275, 1593–1599. 9054347 10.1126/science.275.5306.1593

[B174] Selden N. R. Everitt B. J. Jarrard L. E. Robbins T. W. (1991). Complementary roles for the amygdala and hippocampus in aversive conditioning to explicit and contextual cues. Neuroscience 42, 335–350. 10.1016/0306-4522(91)90379-3 1832750

[B175] Siegel S. Hinson R. Krank M. Mccully J. (1982). Heroin “overdose” death: contribution of drug-associated environmental cues. Science 216, 436–437. 10.1126/science.7200260 7200260

[B176] Sirohi S. Walker B. M. (2015). Maturational alterations in constitutive activity of medial prefrontal cortex kappa-opioid receptors in Wistar rats. J. Neurochem. 135, 659–665. 10.1111/jnc.13279 26257334 PMC4636924

[B177] Skinner B. F. (1948). ‘Superstition’ in the pigeon. J. Exp. Psychol. 38, 168–172. 10.1037/h0055873 18913665

[B178] Smith R. J. Aston-Jones G. (2008). Noradrenergic transmission in the extended amygdala: role in increased drug-seeking and relapse during protracted drug abstinence. Brain Struct. Funct. 213, 43–61. 10.1007/s00429-008-0191-3 18651175 PMC3632504

[B179] Solomon R. L. Corbit J. D. (1978). An opponent-process theory of motivation. Am. Econ. Rev. 68, 12–24.

[B180] Spear N. E. (1978). The Processing of Memories: Forgetting and Retention. Oxford, England: Lawrence Erlbaum.

[B181] Spiteri T. Le Pape G. Ågmo A. (2000). What is learned during place preference conditioning? A comparison of food- and morphine-induced reward. Psychobiology 28, 367–382.

[B182] Spragg D. S. (1940). Morphine addiction in chimpanzees. Comp. Psychol. Monogr. 15, 1–132.

[B183] Staddon J. E. Simmelhag V. L. (1971). The “supersitition” experiment: a reexamination of its implications for the principles of adaptive behavior. Psychol. Rev. 78, 3–43. 10.1037/h0030305

[B184] Steidl S. Wang H. Wise R. A. (2014). Lesions of cholinergic pedunculopontine tegmental nucleus neurons fail to affect cocaine or heroin self-administration or conditioned place preference in rats. PLoS One 9:e84412. 10.1371/journal.pone.0084412 24465410 PMC3897371

[B185] Steiner M. A. Lecourt H. Jenck F. (2013). The dual orexin receptor antagonist almorexant, alone and in combination with morphine, cocaine and amphetamine, on conditioned place preference and locomotor sensitization in the rat. Int. J. Neuropsychopharmacol. 16, 417–432. 10.1017/s1461145712000193 22436395

[B186] Tandon S. Keefe K. A. Taha S. A. (2017). Excitation of lateral habenula neurons as a neural mechanism underlying ethanol-induced conditioned taste aversion. J. Physiol. 595, 1393–1412. 10.1113/jp272994 27682823 PMC5309383

[B187] Thewissen R. Snijders S. J. Havermans R. C. Van Den Hout M. Jansen A. (2006). Renewal of cue-elicited urge to smoke: implications for cue exposure treatment. Behav. Res. Ther. 44, 1441–1449. 10.1016/j.brat.2005.10.010 16375853

[B188] Torregrossa M. M. Taylor J. R. (2013). Learning to forget: manipulating extinction and reconsolidation processes to treat addiction. Psychopharmacology 226, 659–672. 10.1007/s00213-012-2750-9 22638814 PMC3466391

[B189] Torregrossa M. M. Taylor J. R. (2016). Neuroscience of learning and memory for addiction medicine: from habit formation to memory reconsolidation. Prog. Brain Res. 223, 91–113. 10.1016/bs.pbr.2015.07.006 26806773

[B190] Tzschentke T. M. (1998). Measuring reward with the conditioned place preference paradigm: a comprehensive review of drug effects, recent progress and new issues. Prog. Neurobiol. 56, 613–672. 10.1016/s0301-0082(98)00060-4 9871940

[B191] Tzschentke T. M. (2007). Measuring reward with the conditioned place preference (CPP) paradigm: update of the last decade. Addict. Biol. 12, 227–462. 10.1111/j.1369-1600.2007.00070.x 17678505

[B192] Urcelay G. P. Miller R. R. (2008). “1.05—retrieval from memory,” in Learning and Memory: A Comprehensive Reference, ed. J. H. Byrne (Oxford: Academic Press), 53–73.

[B193] Valzachi M. C. Teodorov E. Marcourakis T. Bailey A. Camarini R. (2013). Enhancement of behavioral sensitization, anxiety-like behavior and hippocampal and frontal cortical CREB levels following cocaine abstinence in mice exposed to cocaine during adolescence. PLoS One 8:e78317. 10.1371/journal.pone.0078317 24205196 PMC3804566

[B194] Ventura R. Alcaro A. Puglisi-Allegra S. (2005). Prefrontal cortical norepinephrine release is critical for morphine-induced reward, reinstatement and dopamine release in the nucleus accumbens. Cereb. Cortex 15, 1877–1886. 10.1093/cercor/bhi066 15728739

[B195] Wan L. Xie Y. Su L. Liu Y. Wang Y. Wang Z. (2011). RACK1 affects morphine reward *via* BDNF. Brain Res. 1416, 26–34. 10.1016/j.brainres.2011.07.045 21885037

[B196] Wang B. Luo F. Ge X. C. Fu A. H. Han J. S. (2002). Effects of lesions of various brain areas on drug priming or footshock-induced reactivation of extinguished conditioned place preference. Brain Res. 950, 1–9. 10.1016/s0006-8993(02)02980-3 12231223

[B197] Wang B. Luo F. Zhang W. T. Han J. S. (2000). Stress or drug priming induces reinstatement of extinguished conditioned place preference. Neuroreport 11, 2781–2784. 10.1097/00001756-200008210-00034 10976962

[B198] Wang J. Wu X. Li C. Wei J. Jiang H. Liu C. (2012). Effect of morphine on conditioned place preference in rhesus monkeys. Addict. Biol. 17, 539–546. 10.1111/j.1369-1600.2010.00289.x 21309951

[B199] Wang Y. Zhang H. Cui J. Zhang J. Yin F. Guo H.. (2019). Opiate-associated contextual memory formation and retrieval are differentially modulated by dopamine D1 and D2 signaling in hippocampal-prefrontal connectivity. Neuropsychopharmacology 44, 334–343. 10.1038/s41386-018-0068-y 29728647 PMC6300561

[B200] Wee S. Koob G. F. (2010). The role of the dynorphin-kappa opioid system in the reinforcing effects of drugs of abuse. Psychopharmacology 210, 121–135. 10.1007/s00213-010-1825-8 20352414 PMC2879894

[B201] Wetter D. W. Smith S. S. Kenford S. L. Jorenby D. E. Fiore M. C. Hurt R. D.. (1994). Smoking outcome expectancies: factor structure, predictive validity and discriminant validity. J. Abnorm. Psychol. 103, 801–811. 10.1037/0021-843x.103.4.801 7822583

[B202] Wikler A. (2013). Opioid Dependence: Mechanisms and Treatment. Springer US.

[B203] Wu X. Zhao N. Bai F. Li C. Liu C. Wei J.. (2016). Morphine-induced conditioned place preference in rhesus monkeys: resistance to inactivation of insula and extinction. Neurobiol. Learn. Mem. 131, 192–200. 10.1016/j.nlm.2016.04.005 27101734

[B204] Yan T. Rizak J. D. Wang J. Yang S. Ma Y. Hu X. (2015). Severe dopaminergic neuron loss in rhesus monkey brain impairs morphine-induced conditioned place preference. Front. Behav. Neurosci. 9:273. 10.3389/fnbeh.2015.00273 26528155 PMC4600774

[B205] Yim A. J. Moraes C. R. G. Ferreira T. L. Oliveira M. G. M. (2006). Protein synthesis inhibition in the basolateral amygdala following retrieval does not impair expression of morphine-associated conditioned place preference. Behav. Brain Res. 171, 162–169. 10.1016/j.bbr.2006.03.031 16677727

[B206] Yonghui L. Xigeng Z. Yunjing B. Xiaoyan Y. Nan S. (2006). Opposite effects of MK-801 on the expression of food and morphine-induced conditioned place preference in rats. J. Psychopharmacol. 20, 40–46. 10.1177/0269881105057250 16174676

[B207] Yu L.-L. Wang X.-Y. Zhao M. Liu Y. Li Y.-Q. Li F.-Q.. (2009). Effects of cannabinoid CB1 receptor antagonist rimonabant in consolidation and reconsolidation of methamphetamine reward memory in mice. Psychopharmacology 204, 203–211. 10.1007/s00213-008-1450-y 19148622

[B208] Zachariou V. Bolanos C. A. Selley D. E. Theobald D. Cassidy M. P. Kelz M. B.. (2006). An essential role for DeltaFosB in the nucleus accumbens in morphine action. Nat. Neurosci. 9, 205–211. 10.1038/nn1636 16415864

[B209] Zamble E. Hadad G. M. Mitchell J. B. Cutmore T. R. (1985). Pavlovian conditioning of sexual arousal: first- and second-order effects. J. Exp. Psychol. Anim. Behav. Process. 11, 598–610. 10.1037/0097-7403.11.4.598 4067513

[B210] Zarrindast M. R. Rezayof A. Sahraei H. Haeri-Rohani A. Rassouli Y. (2003). Involvement of dopamine D1 receptors of the central amygdala on the acquisition and expression of morphine-induced place preference in rat. Brain Res. 965, 212–221. 10.1016/s0006-8993(02)04201-4 12591140

[B213] Zhang X.-Q. Cui Y. Cui Y. Chen Y. Na X.-D. Chen F.-Y.. (2012). Activation of p38 signaling in the microglia in the nucleus accumbens contributes to the acquisition and maintenance of morphine-induced conditioned place preference. Brain Behav. Immun. 26, 318–325. 10.1016/j.bbi.2011.09.017 22004988

[B212] Zhang X. Cui Y. Jing J. Cui Y. Xin W. Liu X. (2011). Involvement of p38/NF-κB signaling pathway in the nucleus accumbens in the rewarding effects of morphine in rats. Behav. Brain Res. 218, 184–189. 10.1016/j.bbr.2010.11.049 21130118

[B211] Zhang J. Wang N. Chen B. Wang Y. N. He J. Cai X.. (2016). Blockade of Cannabinoid CB1 receptor attenuates the acquisition of morphine-induced conditioned place preference along with a downregulation of ERK, CREB phosphorylation and BDNF expression in the nucleus accumbens and hippocampus. Neurosci. Lett. 630, 70–76. 10.1016/j.neulet.2016.07.047 27461790

[B214] Zinser M. C. Baker T. B. Sherman J. E. Cannon D. S. (1992). Relation between self-reported affect and drug urges and cravings in continuing and withdrawing smokers. J. Abnorm. Psychol. 101, 617–629. 10.1037/0021-843x.101.4.617 1430600

